# Mechanistic and multiscale aspects of thermo-catalytic CO_2_ conversion to C_1_ products

**DOI:** 10.1039/d1cy00922b

**Published:** 2021-09-30

**Authors:** Md. Imteyaz Alam, Raffaele Cheula, Gianluca Moroni, Luca Nardi, Matteo Maestri

**Affiliations:** Laboratory of Catalysis and Catalytic Processes, Dipartimento di Energia, Politecnico di Milano Via La Masa 34 20156 Milano Italy matteo.maestri@polimi.it

## Abstract

The increasing environmental concerns due to anthropogenic CO_2_ emissions have called for an alternate sustainable source to fulfill rising chemical and energy demands and reduce environmental problems. The thermo-catalytic activation and conversion of abundantly available CO_2_, a thermodynamically stable and kinetically inert molecule, can significantly pave the way to sustainably produce chemicals and fuels and mitigate the additional CO_2_ load. This can be done through comprehensive knowledge and understanding of catalyst behavior, reaction kinetics, and reactor design. This review aims to catalog and summarize the advances in the experimental and theoretical approaches for CO_2_ activation and conversion to C_1_ products *via* heterogeneous catalytic routes. To this aim, we analyze the current literature works describing experimental analyses (*e.g.*, catalyst characterization and kinetics measurement) as well as computational studies (*e.g.*, microkinetic modeling and first-principles calculations). The catalytic reactions of CO_2_ activation and conversion reviewed in detail are: (i) reverse water-gas shift (RWGS), (ii) CO_2_ methanation, (iii) CO_2_ hydrogenation to methanol, and (iv) dry reforming of methane (DRM). This review is divided into six sections. The first section provides an overview of the energy and environmental problems of our society, in which promising strategies and possible pathways to utilize anthropogenic CO_2_ are highlighted. In the second section, the discussion follows with the description of materials and mechanisms of the available thermo-catalytic processes for CO_2_ utilization. In the third section, the process of catalyst deactivation by coking is presented, and possible solutions to the problem are recommended based on experimental and theoretical literature works. In the fourth section, kinetic models are reviewed. In the fifth section, reaction technologies associated with the conversion of CO_2_ are described, and, finally, in the sixth section, concluding remarks and future directions are provided.

## Abstract 2

This review aims to catalog and summarize the advances in the experimental and theoretical approaches for CO_2_ activation and conversion to C_1_ products *via* heterogeneous catalytic routes.

## Graphical abstract

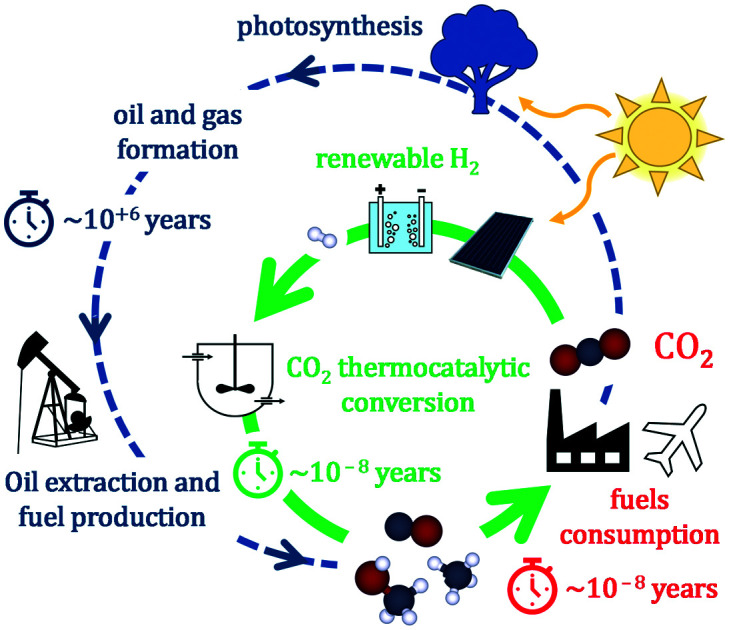

## 1. Introduction

Energy and materials are key requirements of our society and economy. 84% of the primary energy consumed in 2020 was provided by fossil fuels.^1^ Long geological processes (10^6^–10^8^ years) brought about the formation of coal and hydrocarbons from organic materials that originally stored solar energy in their chemical bonds *via* the photosynthesis process from water and CO_2_. Today, while burning hydrocarbons, we make available the energy stored in their chemical bonds with the concomitant re-emission into the atmosphere of the CO_2_ originally consumed in the photosynthesis process. For instance, approximately 22 million tons of coal, 12 million tons of oil (85 million barrels), and 10 billion m^3^ of natural gas are consumed per day to fulfill ∼82% of the total energy demand, resulting in about 30 billion tons of CO_2_ emission every year.^2^ Moreover, today the average power consumption on Earth is expected to increase to 27.6 terawatts by 2050 with an increased population of ∼9.1 billion.^3^ Also, industrial sectors such as steel, iron, and cement industries emit tens to hundreds of tons of CO_2_ annually in a localized and concentrated form for the production of materials essential to our economy. For instance, in 2018 in the European Union, CO_2_ accounted for 81% of total green-house gas emissions, which was 3970 million tons of CO_2_ equivalent, 22.4% of which was from industrial processes, agriculture, and waste management sectors.^4^ As such, all these anthropogenic activities are causing an accumulation of carbon dioxide in the atmosphere.^5^ The average CO_2_ concentration in the atmosphere has been stable for hundred-thousand years in the range of 200–300 ppm, while in the last two centuries it has started to steeply increase, exceeding nowadays 400 ppm.^6,7^ On the one hand, the presence of CO_2_ in the atmosphere is crucial for life on Earth, being involved in the C-cycle and in the regulation of the temperature of the Earth. Without CO_2_, life on Earth would not be possible.^8^ On the other hand, the actual value of the CO_2_ concentration in the atmosphere matters in maintaining the ecosystem compatible with our life. For instance, the increase of the average temperature on Earth causes a wide variety of problems such as more frequent droughts, more intense hurricanes, and the rising sea level.^9–11^ The Intergovernmental Panel on Climate Change (IPCC) has shown a strict link between the rise of CO_2_ concentration in the atmosphere due to anthropogenic activities and the increase of the average temperature on Earth.^6^ Hence, the IPCC stressed that more than 1000 Gt of CO_2_ should not be emitted from 2000 to 2050 to limit the temperature rise to 2 °C.^12^ Moreover, the Paris Agreement in 2015 suggested holding the increasing global temperature below 2 °C compared to preindustrial levels and pursuing efforts to limit the temperature increase to 1.5 °C above the preindustrial level.^12^ Therefore, policymakers are taking action to achieve net-zero CO_2_ emissions. The European Union and other countries such as New Zealand and South Korea are taking action to achieve CO_2_ neutrality by 2050, while China is moving towards the idea of achieving carbon neutrality by 2060. Research and Development (R&D) efforts are required not only to circumvent the CO_2_ problem but to turn it into opportunities by developing resource and energy-efficient processes towards net-zero CO_2_ emissions without compromising the nature, economics, and environment.^13–17^

Solving the problem by eliminating the need for fossil fuels as an energy source is not straightforward since fossil resources are the basis of our current chemical industry.^18^ From a technological point of view, fossil-carbon fuels are also very hard to replace as the main energy vector in our society for three main reasons: (i) they are characterized by huge energy densities; (ii) they are chemically stable under normal conditions; (iii) they are easy to store and transport. A promising concept would be replacing the fossil feedstocks currently used in the chemical and energy industry with sustainably produced chemicals and fuels by reducing CO_2_ using renewable energy. In this view, renewable H_2_ (*e.g.*, water electrolysis with electricity from wind or solar energy) or renewable electricity can be combined with the undesired and highly available CO_2_ for the production of commercially important fuels and chemicals, resulting in a carbon-neutral technology of energy transformation and storage.

Such transformation can be done *via* electrocatalysis, photocatalysis, and thermal catalysis that are essential components in any sustainable energy and chemical production.^19,20^ With the highest oxidized state (+4) and extreme heat of formation (
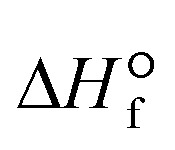
 = −393.5 kJ mol^−1^ at 25 °C), CO_2_ is thermodynamically a highly stable molecule (
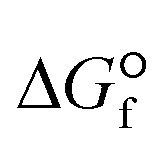
 = −394.4 kJ mol^−1^ at 25 °C), with a very strong C=O bond energy (799 kJ mol^−1^). As a result, the activation and transformation of CO_2_ need very high temperatures or a catalytic process working under suitable operating conditions. For the process of CO_2_ activation and conversion, different technologies have been proposed and developed,^21–38^ including bioconversion (*e.g.* enzyme catalysis),^21,22,28^ electrocatalysis,^31–36^ photochemical reduction,^23–27,37–39^ thermochemical processes,^40–42^ and their combination. This review is focused on thermo-catalytic CO_2_ activation^43–46^ to provide clean, affordable, and secure energy and chemicals by substituting conventional feedstocks.^47^ Furthermore, major attention is paid to the importance of using earth-abundant catalytic materials for CO_2_ activation, which has been widely studied and highlighted in several literature works.^48–54^

State-of-the-art experiments have shown the potential of CO_2_ in making chemical intermediates such as syngas,^55,56^ carbon monoxide,^57^ formic acid,^41^ methanol,^58^ methane,^59^ dimethyl ether,^60^ olefins,^61^ carbamates, carbonates, hydrocarbons (alkanes and aromatics)^62^ and higher alcohols,^63^ which can subsequently be transformed into a myriad of high-value products including chemical process intermediates and fuels. Despite the multitude of fundamental research studies on CO_2_ conversion and its potential to yield important chemical compounds, very few processes have been demonstrated so far with their commercial viability.^64^ The main technological challenges associated with the catalytic conversion of CO_2_ are (i) requirement of significant energy input from carbon-neutral sources to prevent further CO_2_ emission, (ii) need for high temperature and/or pressure processes to achieve good process performances, (iii) requirement of stable and active catalysts to prevent continuous waste of the catalytic material, (iv) poisoning of catalysts by residual water,^65^ (v) sintering of catalyst particles,^66–74^ (vi) coke formation during the reaction,^75,76^ (vii) overoxidation to toxic metal carbonyl (*e.g.*, Ni(CO)_4_),^77–84^ and (viii) waste generation by undesired side reactions.

**Fig. 1:**
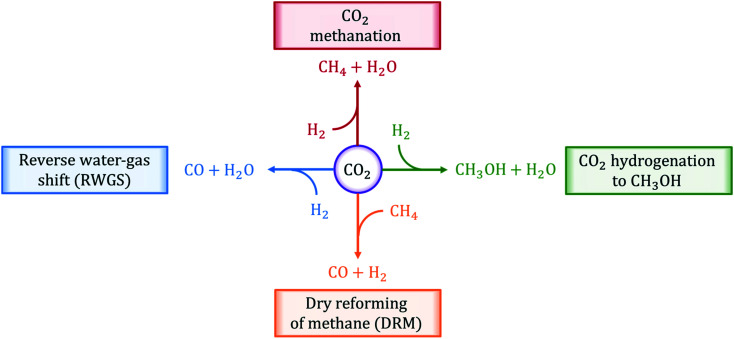
Chemical reactions of thermo-catalytic activation and conversion of CO_2_ to C_1_ products reviewed in this work.

In this review, we present an overview of the state-of-the-art thermally-catalyzed approaches to utilize captured CO_2_ directly by making fuels and chemicals. In particular, we focus on the analysis of four main chemical reactions: (i) reverse water-gas shift (RWGS), (ii) CO_2_ methanation, (iii) CO_2_ hydrogenation to methanol, and (iv) dry reforming of methane (DRM). These reactions are illustrated in Fig. 1. In the analysis and description of the current literature on the selected reaction systems, major attention is paid to (i) catalyst materials employed, (ii) information on the reaction mechanisms gained from experiments and first-principles calculations, (iii) kinetic experiments and corresponding rate equations, (iv) most important challenges and possible solutions, and (v) available reactor technologies.

## 2. Materials and mechanisms

The reaction mechanism of CO_2_ activation and conversion can vary over different catalysts and reaction conditions (*e.g.*, temperature, pressure), and it involves the formation of several different reaction intermediates. The key steps of the reaction scheme are (i) chemical adsorption of CO_2_ and co-reactants (*e.g.*, H_2_, CH_4_), which dissociate over the catalyst surface into reaction intermediates, (ii) surface diffusion and reaction of the intermediates at the catalyst active sites, and (iii) desorption of product species (*e.g.*, CO, CH_4_, CH_3_OH, H_2_O) from the catalyst surface. The information on the reaction kinetics and mechanisms is crucial for the understanding of the pathways of CO_2_ activation and conversion, the identification of the rate-determining steps (RDSs), and the elucidation of the active sites of the catalyst. Such information is fundamental for the optimization of existing processes and the discovery of new catalytic materials, by guiding new experiments or by *in silico* catalyst design. An important concept to take into account when analyzing catalytic systems is that the reaction mechanism and the distribution of the active sites of a catalyst evolve under reaction conditions.^85,86^ Indeed, the local reaction environment that forms during the reaction highly affects the reaction rates,^87^ and catalyst materials can also undergo morphological transformation such as surface reconstruction, phase transition, and deactivation by sintering or coking. These are multiscale phenomena, which need a proper description based on the combination of experimental and theoretical analyses representing the different time and length scales (*i.e.*, from the atomic scale to the reactor scale).

### 2.1 Mechanism of CO_2_ activation and conversion to C_1_ products

**Fig. 2:**
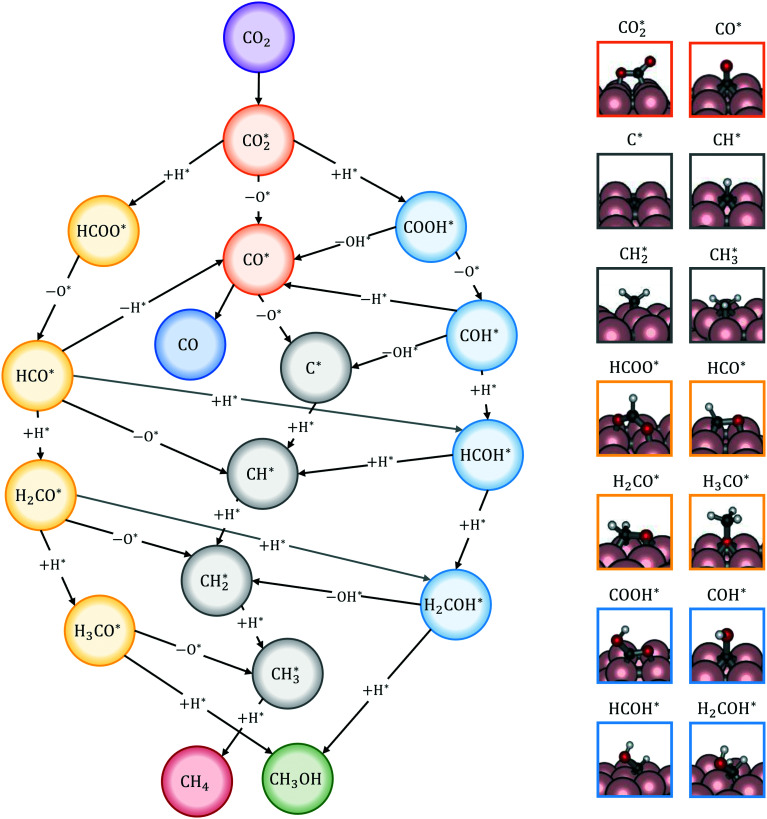
Simplified reaction mechanism of CO_2_ reduction to CO, CH_4_, and CH_3_OH, in which four main reaction paths are highlighted: RWGS redox path (orange), CO* methanation path (grey), formate path (yellow), and carboxyl path (light blue). On the right hand side, the structures of the reaction intermediates^99^ are highlighted with the colors of the corresponding reaction path.

The reactions of transformation of CO_2_ into C_1_ products (CO, CH_4_, and CH_3_OH) proceed through a complex reaction scheme.^88–107^ A simplified version of the scheme is represented in Fig. 2. For illustrative purposes, four main reaction paths^98,99^ are identified in the reaction mechanism and their reaction intermediates are highlighted with different colours. The paths are (i) RWGS redox path, in orange, (ii) CO* methanation path, in grey, (iii) carboxyl path, in yellow, and (iv) formate path, in light blue. In the RWGS redox path, adsorbed 
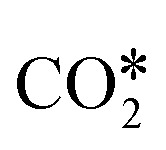
 dissociates into CO* and O*. Then, CO* can desorb as gaseous CO, or it can further react. This path is called “redox” because in RWGS it is accompanied by the oxidation of H* to H_2_O. The dissociation of CO* to C* and O* is the first step of the CO* methanation mechanism, and it is followed by the successive reduction of C* to CH*, 
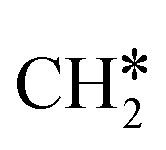
, 
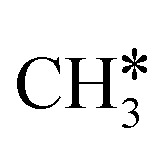
, and gaseous CH_4_, by the addition of H* species. The carboxyl (COOH*) path involves the formation of a COOH* molecule by the addition of an H* to the O atom of 
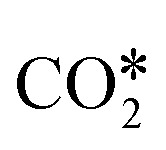
. In the successive steps, COOH* then loses an O* atom, producing COH*, which is successively hydrogenated to HCOH*, and H_2_COH*. The further reduction of H_2_COH* produces CH_3_OH in the gas phase. Similarly, the formate (HCOO*) path involves the reaction of 
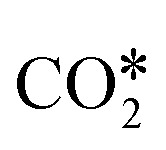
 with an H* atom. However, an HCOO* molecule is formed when H* binds to the C atom of 
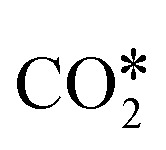
. The formate path continues with the removal of an O* from HCOO* to give HCO*, which is hydrogenated to H_2_CO*, H_3_CO*, and eventually to CH_3_OH. The 
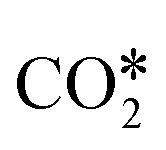
 dissociation, methanation, carboxyl, and formate reaction paths are interconnected by several elementary reactions; indeed, CO and CH_4_ can be formed also from intermediates of COOH* and HCOO* paths. The elementary reactions in the kinetic mechanism involve the addition or the removal of H*, O*, and OH* species. Those adsorbed species are provided by either other elementary steps depicted in Fig. 2 or by the dissociative adsorption of H_2_, and they are precursors for the formation of H_2_O. In DRM, the reaction paths in Fig. 2 which link CH_4_ to CO are followed backwards. Indeed, the adsorption and oxidation of CH_4_ produce CO* and H* species, which are the precursors of syngas (mixture of CO and H_2_). During DRM, carboxyl and formate pathways can also produce CH_3_OH as a side product. Besides the main intermediates and pathways reported in Fig. 2, other reaction intermediates can participate in the overall reaction mechanism. For example, in the carboxyl path, COH* can be formed from COOH* through a 
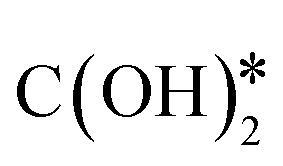
 intermediate. In the formate path, the formation of H_2_CO* from HCOO* can proceed with H_2_COO* or HCOOH* as an intermediate instead of HCO*. Other possible reaction intermediates include 
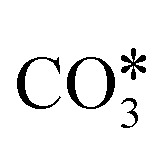
 and species adsorbed onto the catalyst support. Moreover, CO_2_ can also react directly from the gas phase to give HCOO*, COOH*, or CO* and O* species, without being first adsorbed.

In heterogeneous catalysis, CO_2_ activation consists of the interaction of the molecule with a catalytic surface, which usually consists of the chemical adsorption of CO_2_ (the first step of Fig. 2) to give the activated reaction intermediate 
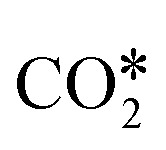
. Usually, the adsorption on the active sites of a surface implies the bending of the CO_2_ molecule. This bending lowers the energy level of the in-plane contribution of the 2π orbital (the lowest unoccupied molecular orbital, LUMO), which makes the carbon atom electrophilic. On metallic surfaces, CO_2_ activation usually consists of electronic charge transfer from the metal to CO_2_.^108–110^ On metal oxides, on the other hand, the adsorption exploits the amphoteric nature of CO_2_, which has a partial positive charge on the C atom (+0.37 e from Mulliken's population analysis) and negative charges on the two O atoms (−0.18 e).^40^ During the adsorption, the electron-deficient C atom and the more electron-rich O atoms can be attacked by either electron-rich or electron-deficient active sites, respectively.^40^ Typically, the carbon atom of CO_2_ interacts through O^2−^ lattice atoms, and the oxygen atoms of CO_2_ interact with the metal cations (*e.g.*, Mn^2+^).

The group of Mpourmpakis studied with DFT the CO_2_ activation on monometallic, core-shell, and decorated icosahedral CuNi (ref. 108) and CuZr (ref. 109 and 110) nanoparticles. For the case of CuNi, it is reported that the presence of surface Ni is key in strongly adsorbing the CO_2_ molecule, which occurs through a charge transfer from the nanoparticles to the CO_2_ molecule, where the local metal d-orbital density localization on surface Ni plays a pivotal role. Additionally, they found a linear relationship between the local-site d-band center and the CO_2_ adsorption energy and observed that the active sites of strong adsorption localize the HOMO (highest occupied molecular orbital) orbitals with increased d-character.^108^ For the case of CuZr nanoparticles, they observed that CO_2_ activation is endothermic on metallic Cu, whereas it becomes barrierless and exothermic on the Zr-decorated Cu nanoparticles, and that the rate of CO_2_ dissociation to CO* and O* is much higher on CuZr than Cu.^109^ Moreover, they showed that Zr sites can be oxidized because of their high oxophilicity; however, they are still able to adsorb and activate CO_2_ easily.^110^ Dixit *et al.*^111^ investigated CO_2_ adsorption on molybdenum carbide (Mo_2_C), showing an important influence of the O* coverage on the CO_2_ adsorption energy and the CO_2_ dissociation barrier. They explained this by showing an electronic modification on the catalyst surface (*e.g.*, d-band shift on Mo atoms) with increasing oxygen coverage.

The adsorption configuration of 
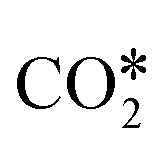
 changes with the structure and composition of the catalyst surface.^99,108–114^ Usually, the C atom and one O atom of 
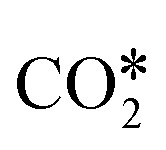
 bind to one or two metal atoms^108–110,112^ (on the “top” or “bridge” adsorption sites). The other O atom of 
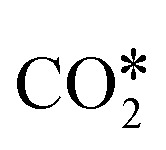
 may interact with the surface.^99,111^ Particularly relevant is the interaction of 
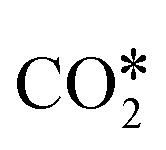
 with the (100) facets of metal catalysts. Indeed, the square geometry of such facets allows for a 
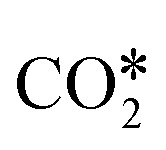
 adsorption configuration in which C and O interact with two metal atoms each, which promotes the breaking of the C=O bond of 
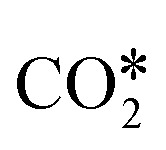
. This yields low activation barriers for 
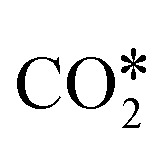
 dissociation to CO* and O* on (100) facets, representing an example of a structure-sensitive elementary step.^112,115^ Regarding the elementary steps needed in the production of hydrocarbons (*e.g.*, CH_4_) from 
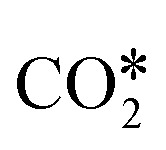
 (Fig. 2), other structure-sensitive effects are reported in the literature.^86,99,116–118^ In those processes, the slow steps are related to the breaking of the triple bond in the CO* molecule (C≡O),^118^ which can occur by direct CO* dissociation^86,116,117^ or by hydrogen-assisted dissociation (*via* a HCO* intermediate).^99,118^ For those reactions, the highest rates are usually provided by the stepped sites available on, *e.g.*, (211),^116,117^ (311),^86,112^ and (110)^99^ facets.

### 2.2 Reverse water-gas shift (RWGS)

RWGS is a reversible and endothermic reaction (Δ*H*^0^_R_ = +41.2 kJ mol^−1^ at 25 °C), in which CO_2_ reacts with H_2_ to give CO and H_2_O.^93,119,120^ It is an equimolar reaction, so its chemical equilibrium is independent of the pressure. At low temperatures, it is usually accompanied by side production of CH_4_*via* CO_2_ methanation.^121,122^ This is because the production of CH_4_ is favored by thermodynamics below ∼400 °C. As a result, high CO selectivity can be achieved by flowing CO_2_ and H_2_ at elevated temperatures.

Several supported metals including Pd,^123,124^ Pt,^100,125–127^ Rh,^128–130^ Au,^122,131,132^ Fe,^133,134^ Ni,^47,94,135^ and Cu,^136,137^ have been reported as active catalysts for the RWGS reaction. In addition, supported metal alloys, such as Pd–In/SiO_2_,^138^ Co–Fe/Al_2_O_3_,^139^ Fe–Cu/Al_2_O_3_,^140^ and Fe–Cu–Cs/Al_2_O_3_,^140^ were employed as catalyst materials, showing high CO selectivity (90–100%) and CO_2_ conversion ranging from 20 to 55%. Among supported metals, high catalytic performances were shown by Cu/CeO_2_ (ref. 137) (25 g_CO_ g_cat_^−1^ h^−1^), Pt/TiO_2_ (ref. 100) (51 g_CO_ g_cat_^−1^ h^−1^), and Rh supported on silicalite-1 (ref. 130) (76 g_CO_ g_cat_^−1^ h^−1^) at 400–450 °C. At the same reaction temperatures, K–Mo_2_C/γ-Al_2_O_3_ (ref. 141) demonstrated also a good catalytic activity (50 g_CO_ g_cat_^−1^ h^−1^), with a catalyst cost much lower than that of noble metals. At a higher temperature (600 °C), Cu/β-Mo_2_C (ref. 136) showed high catalytic activity (40% CO_2_ conversion, 99% CO selectivity) and very good catalyst stability provided by the support that prevents the sintering of Cu nanoparticles.

**Fig. 3:**
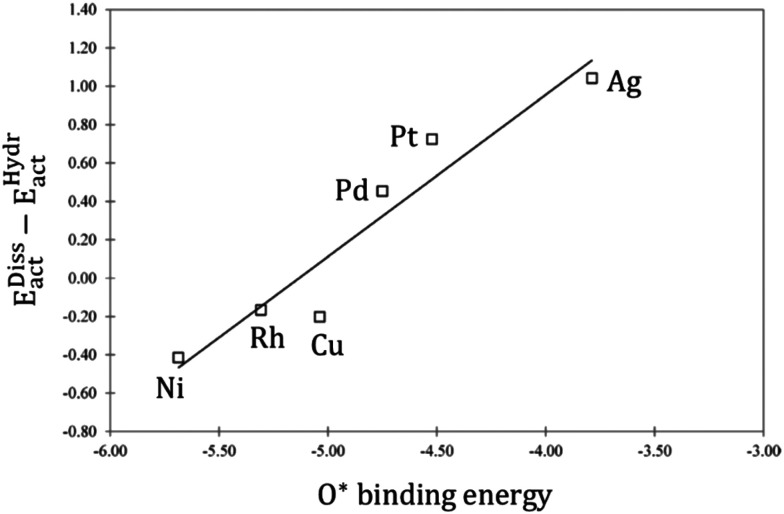
Linear correlation between the O* binding energy and the difference in the activation energies of 
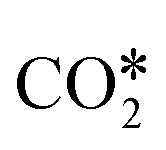
 dissociation (*E*^Diss^_act_) and 
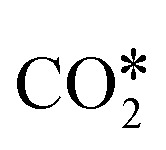
 hydrogenation (*E*^Hydr^_act_). Adapted with permission from ref. 153. Copyright 2015 American Chemical Society.

Based on experimental and theoretical observations, two kinds of mechanisms have been proposed,^125^ namely the redox mechanism^142–144^ and carboxyl (COOH*) mechanism.^145–147^ Formate species, which can form under reaction conditions,^148–150^ are usually reported as spectator molecules that do not participate actively in the reaction. In the redox mechanism, 
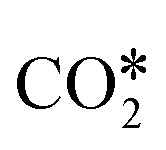
 dissociates to CO* and O*, and the latter reacts with H* (produced by H_2_ adsorption) to give OH* and then H_2_O*.^114^ In the carboxyl mechanism, 
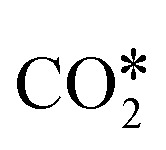
 reacts with H* to give COOH*, which dissociates into CO* and OH*.^151,152^ Maestri and co-workers^153^ studied with DFT calculations the two mechanisms on the (111) surfaces of Pt, Rh, Ni, Cu, Ag, and Pd, showing different reaction paths on the different metals. Indeed, they showed that the redox mechanism is preferred on Rh(111), Ni(111), and Cu(111), whereas the COOH* mechanism is favored on Pt(111), Pd(111), and Ag(111). Brønsted–Evans–Polanyi (BEP) relations for 
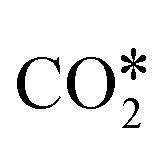
 dissociation and hydrogenation were derived, and the occurrence of different reaction mechanisms was correlated with the oxophilicity of the metals, thus explaining that the stronger the interaction of O* with the metal, the lower the activation energy of 
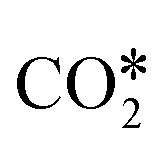
 dissociation, which results in a preferred redox mechanism over the competing COOH* path (Fig. 3).

Several theoretical and experimental observations reported a strong structure-sensitive character of the reactions of CO_2_ conversion,^99,113,154^ including RWGS.^114,155^ For example, Cai *et al.*^113^ investigated the interaction of CO_2_ (and H_2_O) on Ni(111) and Ni(100) surfaces using ambient pressure X-ray photoelectron spectroscopy and theoretical DFT calculations and observed a very different distribution of dissociation products on the two Ni facets in the presence of 0.2 Torr of CO_2_. On Ni(111), more than 90% of the adsorbed surface species are carbonate 
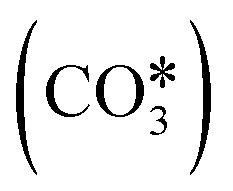
, whereas Ni(100) is mainly covered by adsorbed CO* and graphitic C*. Moreover, they observed with DFT a very high difference in the activation energy of the CO_2_ dissociation reaction on the two facets: 0.33 eV on Ni(100) and 1.34 eV on Ni(111). This is explained by the geometry of Ni(100), which stabilizes the transition state of CO_2_ dissociation by interaction with four Ni atoms interacting with the π orbitals of CO_2_.^115^ Along with this, they showed that the conversion of CO_2_ on Ni(111) and Ni(100) tends to follow different reaction paths, in agreement with their experimental observations. Zhang *et al.*^114^ investigated the RWGS redox mechanism on Ni(111) and Ni(311), showing that the stepped (311) surface has higher catalytic activity than the flat (111) surface. Lin *et al.*^155^ investigated redox and carboxyl mechanisms on Ni(110) in the presence of a high density of subsurface hydrogen, showing that subsurface H atoms can play an important role in RWGS, by both lowering the energy barriers of the kinetic mechanism and participating actively in the hydrogenation elementary steps. They also concluded that, in their system, the redox mechanism is the most favorable RWGS pathway. Wang *et al.*^156^ investigated with transient quantitative temporal analysis of products (TAP) the ability of CO_2_ to re-oxidize a pre-reduced Au/CeO_2_ catalyst. They observed that the reduced catalyst is partially oxidized by CO_2_, suggesting that the redox path is possible on Au/CeO_2_. Wang and Nakamura^157^ performed DFT calculations on different Cu surfaces and revealed a strong structure-sensitivity of 
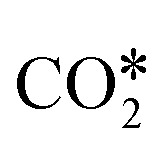
 and H_2_O* dissociation reactions. They calculated activation energies in the following order Cu(110) < Cu(100) < Cu(111) for both elementary dissociation reactions. They reported a “late” nature of the transition states, which resemble the geometry of dissociation products, and they concluded that the activation energies of 
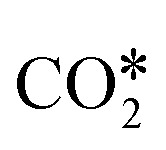
 and H_2_O* dissociation are influenced significantly by the binding energies of OH* and O*, respectively. Liu *et al.*^158^ investigated the redox mechanism on the (100) surfaces of Fe, Ni, Co, and Cu. They reported that Fe(100) shows the highest CO_2_ adsorption energy, which, however, does not facilitate the reaction, but causes a thermodynamic sink on the reaction coordinate. Co(100) and Ni(100), on the other hand, are more favorable in terms of a smaller fluctuation in reaction energies and barriers. They also studied CO_2_ adsorption on Fe bcc(100) and Co hcp(1010) surfaces and suggested that that not only metals but also the surface structures significantly affect the reaction kinetics.

A widely accepted concept is that in the redox mechanism, the RDS is 
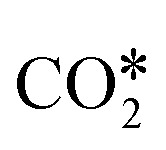
 dissociation.^153,155–158^ In the COOH* mechanism, on the other hand, COOH* formation is usually the limiting step.^153^ The reactions of water formation, *i.e.*, OH* + H* → H_2_O* (and also O* + H* → OH* in the redox mechanism), can also be relevant for the kinetics of the RWGS reaction. Experimental^159,160^ and theoretical^112,161,162^ observations on an Rh/Al_2_O_3_ catalyst suggested that direct WGS and RWGS can follow different reaction mechanisms, where the H_2_O* formation steps are pseudo-equilibrated for RWGS, but they are the slow steps for direct WGS. Maestri and co-workers^161,162^ suggested that the RWGS reaction proceeds through 
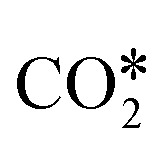
 dissociation over Rh/Al_2_O_3_, whereas direct WGS proceeds with a COOH* intermediate. They calculated that H_2_O* dissociation and 
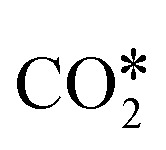
 dissociation are the RDSs for direct and reverse WGS, respectively, in agreement with the experimental reaction orders of Donazzi *et al.*^159,160^ Cheula and Maestri^112^ derived a structure-dependent microkinetic model of direct and reverse WGS on Rh/Al_2_O_3_, which describes the morphological evolution of the catalyst along with the surface reactions on five different Rh facets: (100), (110), (111), (311), and (331). Their study allowed to rationalize that far from equilibrium the two different reacting systems not only follow different reaction pathways but also show that the active sites are different for WGS and RWGS. Indeed, the WGS reaction occurs mainly on the Rh(111) facet, whereas RWGS proceeds on the active sites of Rh(100), which promotes the 
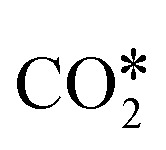
 dissociation elementary step thanks to its structural arrangement.

### 2.3 CO_2_ methanation

CO_2_ methanation, called also the Sabatier reaction,^163^ is an exothermic reaction (Δ*H*^0^_r_ = −165.0 kJ mol^−1^ at 25 °C) favored at low temperatures, in which CO_2_ reacts with 4 molecules of H_2_ to give CH_4_ and 2 molecules of H_2_O. The production of CH_4_ from CO_2_ and H_2_ is advantageous due to the high energy density of CH_4_ and its easiness to be fed into conventional natural gas infrastructure.^164^ Moreover, because of the possibility to convert the Martian CO_2_ atmosphere into methane and water for the fuels and life support of astronauts,^165^ the National Aeronautics and Space Administration (NASA) is spending its efforts to apply this reaction for space colonization on Mars.^165^ The catalysts are typically based on Ni, Ru, Rh, and Co as active phases, with Ni being the most used due to its activity, selectivity, and low cost.^166–172^ The temperature is usually between 100 and 350 °C, as at higher temperatures, the production of CO by RWGS is favored by thermodynamics.^173^

Ni has been the most studied material for industrial CO_2_ methanation applications.^174–176^ High CO_2_ conversion (55–60%) and selectivity to methane (80–90%) were obtained on supported Ni catalysts.^135,177^ However, Ni presents severe deactivation and loss of activity due to carbon formation,^178^ metal sintering,^179,180^ and formation of Ni carbonyls.^181,182^ Alternatively, Rh, Ru, Pd, Pt, and Re on different oxide supports were studied.^130,183–186^ Among them, Ru and Rh showed very high catalytic performances (up to 95% selectivity to CH_4_ and 65–80% CO_2_ conversion).^130,135,187^ Cheaper materials, such as Co,^188,189^ Fe,^190–194^ and Mn,^166,189^ and bimetallic particles containing noble metals, Ni and Fe (ref. 194 and 195), were also investigated.

CO_2_ methanation was reported to be highly dependent on metal–support interaction.^98,196–200^ This phenomenon was related to the ability of CO_2_ to adsorb on the support materials. For instance, Pandey and Deo^194^ studied the reaction on Ni–Fe supported on different materials (*i.e.*, Al_2_O_3_, ZrO_2_, TiO_2_, SiO_2_, and Nb_2_O_5_) and observed that the support yielding the highest activity, Al_2_O_3_, is the one that more favorably adsorbs CO_2_. Martin *et al.*^197^ studied CO_2_ methanation over Rh/CeO_2_ and Ni/CeO_2_ using spectroscopic analyses and demonstrated that Rh/CeO_2_ exhibits higher methane selectivity than the Ni/CeO_2_ catalyst with the same loading. This was explained by the lower Rh particle sizes resulting from the strong metal–support interaction. Moreover, they argued that the CeO_2_ support can influence the reaction rates by getting partially reduced during the reaction and producing Ce^3+^, which can facilitate the activation of CO_2_. Ocampo *et al.*^170^ investigated the Ni/Ce_0.72_Zr_0.28_O_2_ system and reported excellent activity and selectivity of CO_2_ methanation as well as good catalyst stability. The good performances of their catalyst were attributed to the good ability to store active oxygen and to the high Ni dispersibility.^201–203^

**Fig. 4:**
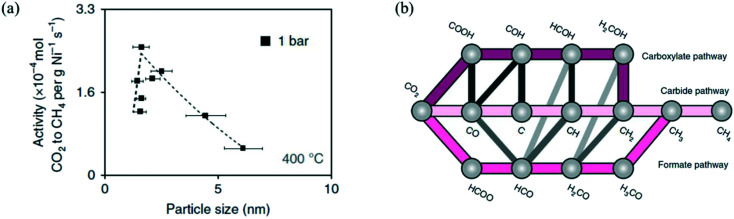
(a) The influence of Ni particle size on activity normalized to the Ni loading for CO_2_ methanation on Ni/SiO_2_, at 400 °C, 1 bar, and H_2_/CO_2_ = 4. Reproduced with permission from ref. 154. (b) Proposed reaction mechanism of CO_2_ methanation on Ni. Reproduced with permission from ref. 99.

The proposed reaction mechanisms of CO_2_ methanation are mainly three: (i) carbide pathway,^204–206^ (ii) COOH* pathway,^207,208^ and (iii) HCOO* pathway,^209,210^ as illustrated in Fig. 4.b. Several authors proposed that a part of the reaction mechanisms can occur on the support^207,211–213^ or at the interfacial sites between the metal and support,^103^ while H_2_ is always supposed to dissociate on the metal. Particularly on the support, the formation of 
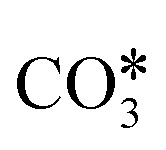
 by the oxidation of CO_2_ is also observed, which can be reduced to bicarbonate 
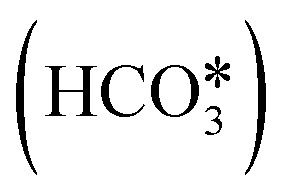
 and participate in the reaction mechanism^211,212^ by producing HCOO* species. Qin *et al.*^213^ achieved high conversion and methane selectivity at 280 °C and over bimetallic catalysts containing Ni, and they suggested bicarbonate, carbonate, and formate as intermediates based on their *in situ* FTIR analyses. Falbo *et al.*^214^ investigated CO_2_ methanation on Rh/Al_2_O_3_ at low and high temperatures and observed higher catalyst stability when CO was added to the inlet. Their spectroscopic analyses were consistent with a path where CO_2_ is adsorbed as bicarbonate on Al_2_O_3_ and it is successively hydrogenated to CH_4_ on Ru, passing through formate and carbonyl intermediates. Aldana *et al.*^207^ studied the reaction on strong and weak basic sites of Ni/Ce_0.5_Zr_0.5_O_2_ and Ni/γ-Al_2_O_3_ and showed that the weak basic sites result in monodentate carbonate, which is more prone to hydrogenation, while the strong basic sites form bidentate carbonates, which do not participate in the reaction mechanism. Yang *et al.*^103^ studied with DFT the CO_2_ methanation over a cluster of Rh supported on TiO_2_, highlighting the role of the metal–support interface, which is a significant charge accumulation region and can provide electrons for CO_2_ reduction. In their work, they showed that the interface active sites are more feasible for CO_2_ activation than the Rh nanoparticle sites. Moreover, they calculated that the COOH* mechanism involving the formation of a COH* intermediate is the favorite reaction pathway of CO_2_ methanation over the Rh/TiO_2_ catalyst, while the redox WGS and the formate pathways show higher energy barriers. Such preferred pathways proceed with the intermediates 
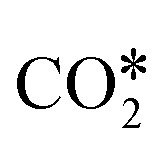
, COOH*, CO*, COH*, HCOH*, H_2_COH*, 
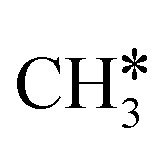
, and 
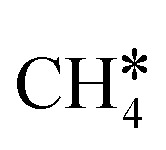
, and show COOH* dissociation into CO* as the RDS.

Vogt *et al.*^99,154^ highlighted the strong structure-sensitive character of CO_2_ methanation over Ni catalysts. They studied the effect of a Ni/SiO_2_ nanocluster with particle size 1–7 nm and observed an optimum in the CO_2_ methanation activity at a particle size of 2–3 nm (Fig. 4.a).^154^ To understand such a particle size effect, they also studied CO_2_ methanation on Ni catalysts supported on different metal oxide supports (Al_2_O_3_, CeO_2_, ZrO_2_, TiO_2_), and they developed a microkinetic model based on DFT calculations on the (100), (110), (111), and (211) facets of Ni (reaction mechanism shown in Fig. 4.b). The integration of their microkinetic model allowed them to select the most active Ni facet, *i.e.*, Ni(110), and identify the dissociation of HCO* (HCO* → CH* + O*) on such a facet as the RDS of the reaction mechanism. The maximum in the activity *vs.* size plot was explained by two phenomena: (i) the catalytic activity decreases with the particle size because big particles have a lower dispersion (*i.e.*, the fraction of metal atoms at the surface) and therefore a lower number of active sites per gram of catalyst, and (ii) particles with size lower than 2–3 nm have low turnover frequency (TOF), (*i.e.*, the catalytic activity per active site) because they do not show the most active sites for the reaction.^215^

The main phenomena that determine catalyst deactivation during CO_2_ methanation are sintering and coking. To identify the nature of carbon formed during methanation reactions over Ni/Al_2_O_3_, Olesen *et al.*^216^ performed temperature-programmed hydrogenation experiments and observed three major carbon peaks, attributed to carbide (∼650 K) and polymeric carbon (∼650 and ∼775 K). Loss of activity with an increase in the carbon-to-nickel ratio was noticed, primarily due to polymeric carbon formation that resulted in catalyst deactivation. A linear correlation was observed between the amount of carbon formed and the degree of deactivation of the catalyst.^216^ Barrientos *et al.*^217^ investigated various Ni/Al_2_O_3_ catalysts promoted with MgO, CaO, BaO, and ZrO_2_ under low-temperature conditions (300 °C), and they showed that the presence of Zn lowers the formation of polymeric carbon. Galhardo *et al.*,^92^ with *in situ* spectroscopic analyses, observed that the accumulation of carbon species on the surface of a Ni/SiO_2_ catalyst at high temperatures leads to a Ni_3_C-like phase, which changes the process selectivity towards the formation of CO. After carbon depletion from the surface of the Ni particles by oxidation, the catalyst regains its high selectivity to CH_4_ production. However, the selectivity readily shifts back toward CO formation after exposing the catalysts to a new temperature-programmed CO_2_ hydrogenation cycle. The fraction of weakly adsorbed CO* increases on the Ni_3_C-like surface when compared to a clean nickel surface, explaining the higher selectivity to CO.

### 2.4 Dry reforming of methane (DRM)

Syngas (a mixture of CO and H_2_) is an important intermediate used as a building block molecule to manufacture high-value chemicals (*e.g.*, methanol, olefins, and hydrocarbons), and it can be produced from CO_2_*via* DRM,^218,219^ a reaction in which CO_2_ combines with CH_4_ to give 2 molecules of CO and 2 molecules of H_2_. As DRM is an endothermic reaction (Δ*H*^0^_R_ = +247.0 kJ mol^−1^ at 25 °C), it is thermodynamically favorable at high temperature, so the reaction temperature is usually between 550 and 800 °C. On the surface of a catalyst, CO_2_ and CH_4_ dissociate into reaction intermediates (*e.g.*, CO*, O*, H*, OH*, 
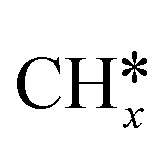
, 
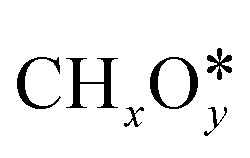
), which combine to give the final reaction products (CO and H_2_). The most relevant unwanted side-product is coke, which can accumulate and deactivate the catalyst.

Nickel is the most important and utilized material for DRM, because of its high activity, high abundance, and low cost. High conversion of both CO_2_ and CH_4_ (80–95% at 700–800 °C) was obtained with Ni catalysts supported on different materials such as Al_2_O_3_,^220^ SiO_2_,^221^ and BN.^222^ However, rapid coke deposition on the catalyst surfaces during the reaction limits the practical use of Ni-based catalysts.^217,223^ To cope with such challenges, noble metals like Rh Ru, Pt, Ir, and Pd were also investigated, because of their higher coking resistance.^224–226^ Good catalyst performances and resistance to deactivation by coking were showed by supported bimetallic catalysts such as Ni–Rh/LaAlO_3_,^227^ Ni–Cu/MgO,^228^ and Ni–Mn/Al_2_O_3_.^229^ Such hybrid materials have a lower cost than noble metal catalysts and show high catalytic performances (85–97% CO_2_ and CH_4_ conversion at 750–800 °C).^227–229^

**Fig. 5:**
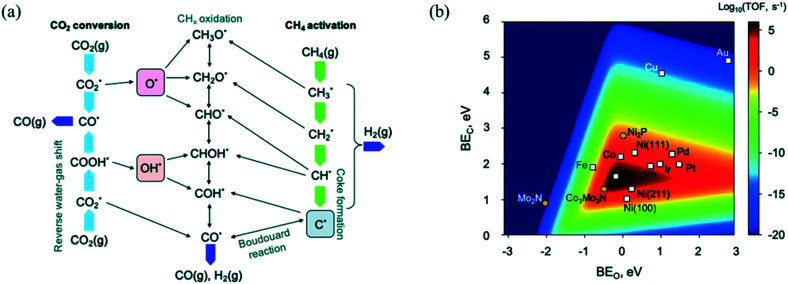
(a) DRM reaction network displaying the conversion routes of CH_4_ (green arrows) and CO_2_ (light blue arrows), including relevant reaction intermediates and elementary steps. (b) 2D volcano plot indicating the DRM catalytic activity (TOF) as a function of the binding energies of C* and O*. Adapted with permission from ref. 242.

Several researchers have attempted to understand the mechanism of DRM on supported metal catalysts.^230–235^ Bradford *et al.*^235^ performed kinetic studies on Ni supported on different materials (SiO_2_, TiO_2_, MgO, and C) and suggested a reaction mechanism consistent with their experimental data. In their mechanism, CO_2_ dissociates to CO* and O*, and CH_4_ dissociates into 
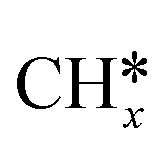
 and H*. Then, OH* species are formed and act as an oxidant. Indeed, 
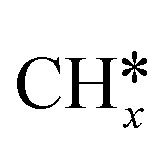
 gets oxidized by OH* to form CH_*x*_OH* species, which produce CO and H_2_ upon subsequent reactions. Both CH_4_ dissociation and CH_*x*_OH decomposition were supposed as the slow steps of the mechanism. Múnera *et al.*^236^ have concluded in their kinetic studies of DRM on Rh/La_2_O_3_–SiO_2_ that CH_4_ decomposition takes place on the metal, whereas CO_2_ activation occurs on the support. Maestri *et al.*^237^ proposed CH_4_ dissociation (CH_4_ → 
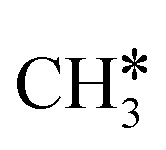
 + H*) as the RDS on an Rh/Al_2_O_3_ catalyst, from hierarchical microkinetic modeling. With their analysis, they show that CH_4_ dehydrogenates into atomic carbon (C*), which gets oxidized subsequently by OH* over Rh surfaces. Mark *et al.*^238^ concluded that CH_4_ dissociation is the RDS for DRM also over Ir/Al_2_O_3_ at 700–850 °C and 1 atm. However, they reported that adsorbed carbon C* reacts *via* the reverse Boudouard reaction (BR) (C* + 
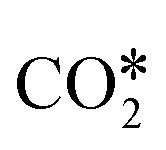
 ⇌ CO*) to give the final products.^239^ CH_4_ dissociation was further confirmed as the RDS in DRM by Iglesia and co-workers through conducting experiments over supported Ni, Pd, and Rh catalysts at temperatures lower than 600 °C using the CH_4_/CD_4_ isotope tracer method.^239–241^ Fan *et al.*^230^ derived a comprehensive microkinetic model based on DFT calculations on Ni(111), Ni(211), and Ni(100) facets, and showed that at low CH_4_ and CO_2_ partial pressures, both CH_4_ dissociative adsorption and C* oxidation affect the overall reaction rate, whereas, at high pressures, C* oxidation is suggested as the only RDS for the DRM reaction, in agreement with previous experimental observations. Manavi and Liu^242^ investigated the reaction mechanism of DRM on the (111) facet of Co_3_Mo_3_N, a catalyst that can activate easily both C=O and C–H bonds, and proposed a reaction network for DRM, as shown in Fig. 5.a. Moreover, they derived a 2D volcano plot illustrating the catalytic activity *vs.* the binding energy of C* and O* for different materials (shown in Fig. 5.b), highlighting the importance of mild adsorption energies for the highest catalytic activity.

Since carbon is miscible in Ni surfaces, it can diffuse through the surface and form carbide-like phases such as Ni_3_C.^243,244^ To understand the role of Ni_3_C in syngas production and catalyst deactivation, Wang *et al.*^245^ investigated DRM over flat and stepped Ni and Ni_3_C surfaces with DFT calculations. They reported that, due to the high CH oxidation rate and low CO dissociation, the flat Ni(111) surface shows high catalytic activity for DRM and low coke formation. The flat Ni_3_C(001) shows good DRM catalytic activity, but it also produces coke. The stepped surfaces of both nickel – Ni(211) – and nickel carbide – Ni_3_C(111) – show instead poor DRM performances and high coke formation.

The support material is another important factor that can modify the reactivity and stability of the catalyst under reaction conditions by changing its dispersion and its electronic properties.^246^ The role of different supports and additives in coke deposition and the durability of Ni-based catalysts has been extensively studied.^194,247–249^ Sokolov *et al.*^250^ investigated the role of different supports in the activity of Ni at 400 °C. Their Ni/La_2_O_3_ZrO_2_ catalyst was reported to be the most active and most stable. They also studied different structures of La_2_O_3_ZrO_2_, *i.e.*, nonstructured, mesoporous, and microporous. The mesoporous support showed no change in activity over 180 h on-stream, whereas the others deactivated, by the formation of graphene-like coke layers and NiO shells over Ni particles. The enhanced stability of Ni on the mesoporous La_2_O_3_ZrO_2_ was attributed to a pore confinement effect. Yavuz and co-workers^218^ demonstrated very high stability of the Ni–Mo/MgO catalyst (up to 850 h) without coke formation at 800 °C under DRM conditions. This high stability was presumed to be due to the small particle size (2.9 nm) of the Ni–Mo/MgO nano-catalyst that may lead to better dispersibility and prevent coke formation.

### 2.5 CO_2_ hydrogenation to methanol

Methanol is an important chemical compound that can be used as an additive for fuels or as a precursor of, *e.g.*, dimethyl ether, gasoline, and diesel. CO_2_ hydrogenation to methanol is an exothermic reaction (Δ*H*^0^_r_ = −49.5 kJ mol^−1^ at 25 °C), favored at low temperatures and high pressures. A good catalyst for the production of methanol from CO_2_ must have active sites for the activation of CO_2_, dissociate H_2_, and suppress competitive side reactions (*e.g.*, RWGS, methanation).^251–254^ Usually, this implies the use of active metal-oxide materials,^52,253^ which can also be doped to change their surface electronic properties.^255–258^

Industrially, methanol is produced from synthesis gas mixtures (CO/CO_2_/H_2_) at 200–300 °C and 50–100 bar (ref. 259) over Cu/ZnO/Al_2_O_3_ catalysts, selected because of the low cost of Cu and the good synergism between Cu and ZnO. For CO_2_ conversion, the Cu/ZnO/Al_2_O_3_ catalyst displays lower activity towards methanol formation due to the competitive RWGS reaction^260^ and water-induced deactivation.^261,262^ The production of H_2_O is higher when CO_2_ is present in the feed because the CO_2_ to methanol reaction stoichiometry (CO_2_ + 3H_2_ ⇌ CH_3_OH + H_2_O) implies the formation of an H_2_O molecule for every CO_2_ molecule.^261–263^ The production of H_2_O enhances also the sintering of the catalyst.^70–74,263^ To overcome these challenges, different support materials, which can promote structural and electronic properties and stabilize smaller Cu particles,^264^ were investigated, including ZrO_2_,^265,266^ La_2_O_3_,^267^ MoC_2_,^267^ CeO_2_,^268^ and La_2_O_2_CO_3_ (ref. 267) and ZnO–ZrO_2_.^98,259,269–275^ In particular, Cu/ZnO/ZrO_2_ showed very high activity and selectivity at low temperatures (180–240 °C).^276–279^ This is likely due to the weak hydrophilic nature of ZrO_2_, which may inhibit the poisoning effect of water on the active sites^272,273^ and promote the activity by increasing Cu dispersion, which can strongly affect CO_2_ adsorption and methanol selectivity.^280,281^ Other materials investigated for CO_2_ hydrogenation to methanol are Au/CeO_*x*_/TiO_2_,^282^ In_2_O_3_,^283–287^ Ir–In_2_O_3_,^288^ Ni–In_2_O_3_,^289^ Mn–Co,^290^ Ni–Ga,^291^ ZnO–ZrO_2_,^292^ GaPd_2_,^293^ and Co/SiO_2_.^91^ In particular, In_2_O_3_ is attracting great interest in the scientific community^283–287^ because of its high catalyst stability^265,286^ and very high methanol selectivity.^294–298^ However, there is still a debate on the currently available best catalyst of methanol synthesis from CO_2_, mainly because the performances of the catalysts strongly depend on the experimental conditions under which they are tested. An In_2_O_3_/ZrO_2_ catalyst^287^ provided high methanol selectivity (99.8%), low CO_2_ conversion (5.2%) and a productivity of 0.30 g_MeOH_ g_cat_^−1^ h^−1^ at 300 °C, 50 bar, with a gas hourly space velocity (GHSV) of 16 000 h^−1^. Under similar experimental conditions (240 °C and 50 bar), a Cu/ZnO/ZrO_2_ catalyst showed higher methanol productivity (1.2 g_MeOH_ g_cat_^−1^ h^−1^), a CO_2_ conversion of 9.7% and a methanol selectivity of 62.0%.^299^ At 280 °C, 46 bar, and GHSW = 10 000 h^−1^, Cu/ZnO/Al_2_O_3_ (ref. 300) showed higher CO_2_ conversion (23.8%) but lower methanol selectivity (22.8%) and a productivity of 0.15 g_MeOH_ g_cat_^−1^ h^−1^. The same Cu/ZnO/Al_2_O_3_ catalyst at extreme pressure (442 bars) showed much better performances, *i.e.*, a CO_2_ conversion of 84.7%, a methanol selectivity 93.1% and a productivity of 2.18 g_MeOH_ g_cat_^−1^ h^−1^,^300^ highlighting the strong positive effect of the pressure on the thermodynamics and kinetics of the reaction.

**Fig. 6:**
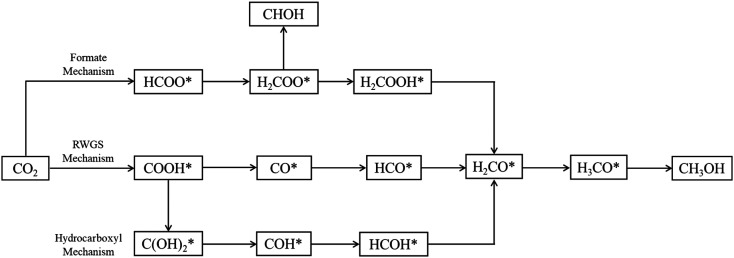
Proposed reaction paths^301^ of methanol synthesis *via* CO_2_ hydrogenation over Cu-based catalysts.

Three main pathways^301^ have been proposed for CO_2_ hydrogenation to methanol (Fig. 6), with a mechanism slightly different from the one represented in Fig. 2. In the formate (HCOO*) mechanism,^259,302,303^
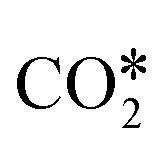
 is hydrogenated to HCOO*, H_2_COO* (or HCOOH*), and to H_2_COOH*. Then, an OH* is removed, yielding H_2_CO*, which is hydrogenated to H_3_CO* and CH_3_OH. In the RWGS mechanism, CO* is formed from 
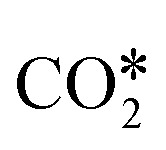
 through a COOH* intermediate, then CO* is hydrogenated to HCO*, H_2_CO*, H_3_CO*, and CH_3_OH. In the hydroxycarbonyl mechanism, COH* is formed through a 
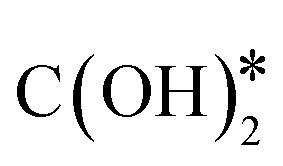
 intermediate, and then it is hydrogenated to HCOH*, H_2_CO*, H_3_CO*, and CH_3_OH.

**Fig. 7:**
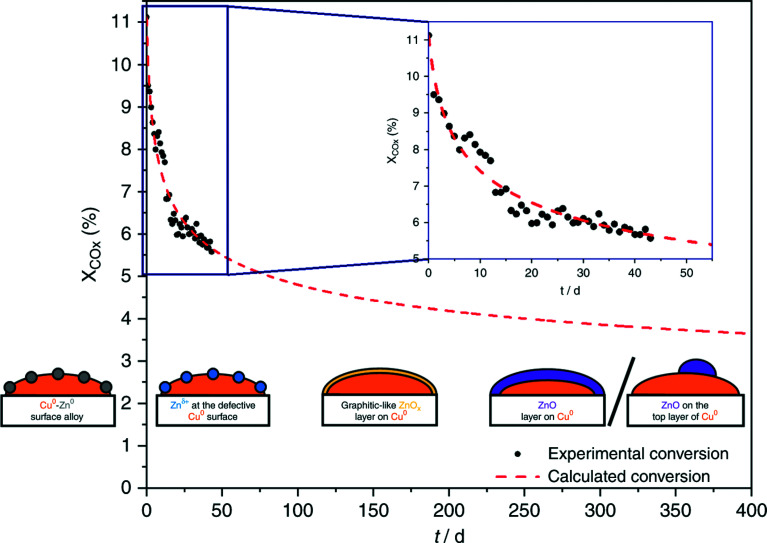
Long-term methanol production over the industrial Cu/ZnO/Al_2_O_3_ catalyst at 210 °C and 60 bar, under different controlled conditions. Black points refer to the recorded degrees of conversion of the reaction; the dashed red line describes the intra- and extrapolation of the experimental data. Illustrations from right to left: Cu^0^–Zn^0^ surface alloy, Zn^*δ*+^ species at the defective Cu^0^ surface, and graphitic-like ZnO_*x*_ layer on Cu^0^. ZnO layer on Cu^0^ and ZnO on the top layer of Cu^0^. Reproduced with permission from ref. 307.

Regarding the Cu/ZnO system, many hypotheses on the nature of the active sites were proposed.^259,304,305^ Fujita *et al.*^306^ investigated the system with diffuse reflectance FT-IR spectroscopy and temperature-programmed desorption, showing that two types of formate species and zinc methoxide – Zn(CH_3_O)_2_ – form during the reaction. Zinc methoxide was readily hydrolyzed to methanol, whereas H_2_O formed through RWGS was suggested to be involved in the hydrolysis of zinc methoxide. In a recent communication, Muhler and co-workers^307^ investigated CO_2_ hydrogenation to methanol on Cu/ZnO/Al_2_O_3_ with a surface-sensitive *operando* method using a high-pressure pulse experiment (HPPE) and proposed a mechanism for long-term catalyst deactivation, as illustrated in Fig. 7. In the beginning, reduced ZnO_*x*_ species migrate onto the metallic Cu^0^ nanoparticles and form Cu^0^–Zn^0^ alloys.^305^ Then, under CO_2_ hydrogenation, different oxygen-containing adsorbates form, which partially oxidize Zn^0^ to Zn^*δ*+^ at the defective Cu^0^ site.^259^ The subsequent migration of Zn species leads to a graphitic-like ZnO_*x*_ layer on Cu^0^ surfaces,^304^ which ultimately turns into a stable and crystalline layer of ZnO,^304^ covering the Cu^0^ surface completely or partially.^269^ During the process, the highly active Cu^0^–Zn^*δ*+^ sites were found embedded in a constantly changing matrix provided by the Cu/ZnO/Al_2_O_3_ catalyst.

The reaction mechanism and RDS for methanol production over Cu are still under debate.^308–312^ Bowker *et al.*^310^ suggested the hydrogenation of H_2_CO* to H_3_CO* as the rate-limiting step. Other authors^308,309^ reported instead the hydrogenation of HCOO* to H_2_COO* as the RDS over Cu(111). Zhao *et al.*^311^ showed that the HCOO* path is kinetically unfavorable compared to the COOH* route, especially in the presence of water. The isotope labeling experiment by Chinchen *et al.*^313^ suggested that CO may not be an essential intermediate for methanol formation from CO_2_. Mavrikakis and co-workers^312^ produced a microkinetic model based on DFT calculations which describes WGS and methanol synthesis from CO and CO_2_ on Cu(111) and highlighted the role of HCOOH* as a reaction intermediate. They showed different sequences of intermediates in methanol production from CO (HCO*, CH_2_O*, and CH_3_O*) and CO_2_ (HCOO*, HCOOH*, 
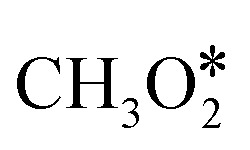
, CH_2_O*, CH_3_O*). They show that methanol synthesis rates are limited by methoxy (CH_3_O*) formation in CO-rich environments and by CH_3_O* hydrogenation in CO_2_-rich feeds. Higham *et al.*^314^ have concluded in their DFT studies that Cu(110) and Cu(100) are more active facets for CO_2_ dissociation and hydrogenation than the more abundant Cu(111) surface. Moreover, they showed that the synthesis of methanol on those Cu surfaces can follow also reaction paths with COOH* as an intermediate. Eventually, some experimental observations suggest that the formate mechanism should not be the preferred one, because (i) direct hydrogenation of formate on the Cu surface did not result in methanol in the absence of water;^309^ (ii) the experimentally observed formate hydrogenation kinetics was inconsistent with that of methanol formation,^308^ and (iii) during methanol synthesis, formaldehyde, easily produced from H_2_CO*, has not been detected as a side product.^315,316^ Therefore, further theoretical studies, especially on the study of the active sites at the catalyst–support interface, are required.

Recent experimental studies demonstrated high performances of In_2_O_3_ catalysts for methanol synthesis from CO_2_.^283–287^ Martin *et al.*^287^ produced an In_2_O_3_/ZrO_2_ catalyst showing 100% methanol selectivity at 200–300 °C and 1–5 MPa and high stability (1000 h on stream). The excellent selectivity of the catalyst was attributed to the formation of oxygen vacancies, promoted by both the ZrO_2_ support and a co-feeding of CO. The group of Nørskov^317^ investigated with DFT the methanol synthesis on In_2_O_3_(111) and In_2_O_3_(110). They produced a theoretical volcano plot illustrating a clear relationship between the number of reduced surface In layers and the catalytic activity of In_2_O_3_(111). Moreover, they explained the positive effect on the catalytic activity of the ZrO_2_ support, which influences the number of reduced In layers.

Following these findings, Zhou *et al.*^318^ investigated with DFT analyses the reaction mechanism on In_2_O_3_(111) and In_2_O_3_(110). They reported that CO production from CO_2_*via* both redox and COOH* routes is kinetically slower than methanol formation under typical steady-state conditions, in agreement with the experimental observations showing high methanol selectivity of In_2_O_3_. They reported a formate mechanism of methanol synthesis in which H_2_COO* is produced from HCOO* and then dissociates to H_2_CO* and O*. The O* fills the oxygen vacancy site of In_2_O_3_, and H_2_CO* is then hydrogenated to H_3_CO* and CH_3_OH. Their calculated RDS is the homolytic H_2_ dissociation and suggested that the reaction rate can be enhanced by introducing transition metal dopants which speed up H_2_ dissociation. Dang *et al.*^319^ investigated with DFT the cubic and hexagonal surfaces of In_2_O_3_, showing that the hexagonal In_2_O_3_(104) shows far superior catalytic performance. Moreover, they synthesized a novel hexagonal In_2_O_3_ nanomaterial that exhibits very high activity, methanol selectivity, and catalytic stability. On In_2_O_3_, the formate mechanism is suggested as the most probable pathway, entailing cyclic creation and annihilation of oxygen vacancy active sites, and involving the reaction intermediates HCOO*, H_2_COO*, H_2_CO*, and H_3_CO*.^318^

A major shortcoming of In_2_O_3_ catalysts is the long-term stability. To investigate morphological changes of the In_2_O_3_ catalyst during CO_2_ hydrogenation to CH_3_OH, Tsoukalou *et al.*^285^ performed combined time-resolved *operando* XAS–XRD and *in situ* TEM experiments. Their results show the reduction of In_2_O_3_ during the reaction by the formation of oxygen vacancy sites (In_2_O_3−*x*_), followed by the amorphization of In_2_O_3_ nanoparticles into a dynamic mixture of In^0^/In_2_O_3−*x*_, in which crystalline and amorphous phases coexist and continuously interconvert. The formation of metallic In^0^ was reported to cause the deactivation of the catalyst.

## 3. Carbon formation and design for coke-tolerant catalysts

Catalyst deactivation due to carbon formation is one of the critical technological problems for CO_2_ conversion processes.^66,223^ It is an inevitable process that can lead to a temporary or permanent catalyst deactivation *via* pore blockage,^320,321^ metal particle encapsulation,^322^ or breakage of the catalyst pellets.^217^ In the processes reviewed in this work, carbon is primarily produced by CO disproportionation (2CO → C* + CO_2_) and CH_4_ dehydrogenation (CH_4_ → C* + 2H_2_). The latter reaction is favored at high temperature, while CO disproportionation is exothermic and thus thermodynamically favored at a temperature lower than 500 °C.

The carbon formed at the catalyst surface can assume various forms depending on the operating conditions,^323–325^ each form exhibiting unique features and different reactivities.^244,326^

**Fig. 8:**
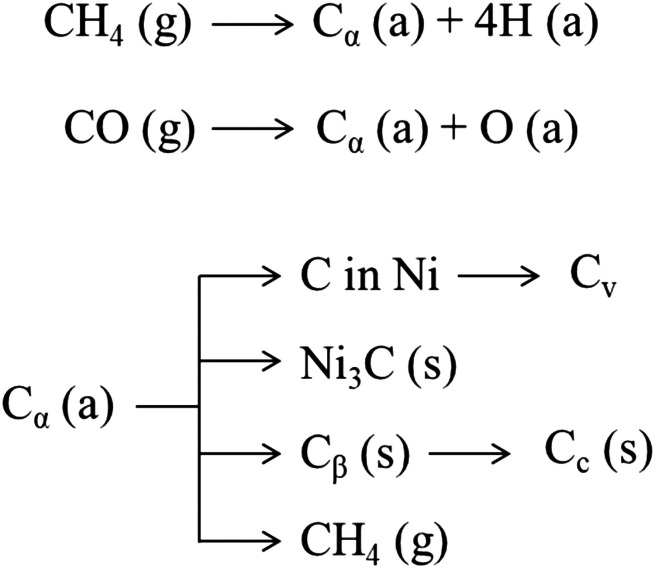
Various forms of carbon^327^ generated from CO over the Ni surface. (a), (g), and (s) refer to adsorbed, gaseous, and solid states, respectively.

As shown in Fig. 8, CO and methane dissociate on the metal surface to form a highly reactive carbon species, C_α_ (probably an adsorbed atomic carbon), which can be gasified (to CH_4_) or turned into the less reactive C_β_ and assume the form of polymeric carbon films or filaments.^327^ On exposure to high temperature (*T* > 500–550 °C), amorphous carbon (C_β_) is transformed into an even less reactive graphitic form (C_c_).^327^ Some of these carbon structures such as polymeric (C_β_) and graphitic (C_c_) films are responsible for deactivation due to encapsulation of the metal particles at the catalyst surface.^328^ Meanwhile, other forms such as filamentous carbon, generally do not deactivate metal surfaces but may plug catalyst pores and break up catalyst pellets.^328^ The growth of such carbon species depends on the possibility and the ease of the carbon C_α_ to dissolve through the metal particles.^329^ The dissolved carbon can diffuse through the metallic crystallite to nucleate and precipitate at the rear of the particles.^244^ This process leads to the formation of carbon whiskers, which lift the crystallite from the support surface and eventually result in the fragmentation of the catalyst.^328^ The driving force for intra-particle carbon diffusion and whisker growth is a gradient in carbon activity (*i.e.*, concentration) across the metal particle.^244^ Noble metals, except for palladium at 650 °C, do not form this carbon structure; the structure of the carbon formed on noble metals is difficult to distinguish from the catalyst structure, with few atomic layers of carbon covering almost completely the surface.^330^ Leung *et al.*^331^ proposed that carbon formation rates and morphologies on Ni (*i.e.*, filamentous or encapsulating) are solely determined by the pressure ratio *P*_CH_4__*P*_CO_/*P*_CO_2__, which sets the thermodynamic carbon activity at the metal surface. Low values of this ratio lead to the formation of carbon filaments with a rate proportional to the ratio itself. Meanwhile, high values of carbon activity lead to the simultaneous nucleation of multiple carbon patches, with the consequent formation of carbon adlayers that brings about the encapsulation of Ni nanoparticles. In this sense, carbon formation rates decrease with decreasing metal particle size because of the lower stability and concomitant increase in activity of the smaller diameter carbon filaments formed on smaller metal nanoparticles. This phenomenon was reported also by other authors.^332,333^

Under common industrial operative conditions (*T* > 800 °C), CH_4_ decomposition is the main cause of carbon formation during DRM.^333^ For a given CO_2_/CH_4_ ratio, the temperature below which carbon deposits are formed decreases as the pressure decreases, while at constant pressure, this temperature limit increases as the CO_2_/CH_4_ ratio decreases.^334,335^ Thus, working with an excess of CO_2_ in the feed may reduce carbon formation at lower temperatures. With stoichiometric feeds, a temperature higher than 800 °C should suppress carbon deposits where there is thermodynamic potential.^224^ Catalyst deactivation due to carbon formation was observed during RWGS only when the reaction is conducted at low temperatures. Goguet *et al.*^336^ observed a slow but continued catalyst deactivation due to carbon formation over 2% Pt/CeO_2_ under RWGS conditions at 300 °C mainly due to CO disproportionation. Witte *et al.*^337^ experienced a loss in activity of a Ni catalyst during an 1100 h test at 350 °C under CO_2_ methanation conditions. They proved the presence of carbon deposits through TPO with oxygen conducted over the spent Ni-based catalyst and suggested the addition of steam into the reactor feed to prevent coking. Carbon deposits were also proved to form under the same conditions over noble metals catalysts by Solymosi *et al.*^171,338,339^

**Fig. 9:**
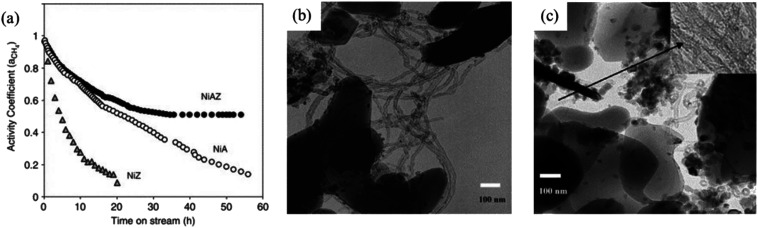
(a) Catalyst deactivation in terms of the CH_4_ activity coefficient (ratio between the CH_4_ consumption rate and the initial consumption rate) during DRM over Ni-αAl_2_O_3_ (NiA), Ni–ZrO_2_ (NiZ), and Ni–ZrO_2_–Al_2_O_3_ (NiAZ) catalysts at 700 °C. (b) TEM image of the Ni–αAl_2_O_3_ catalyst after DRM deactivation at 700 °C. (c) TEM image of the Ni–ZrO_2_–Al_2_O_3_ catalyst after the DRM deactivation test at 700 °C. Adapted with permission from ref. 340.

Once formed, carbon may be removed from the catalyst surface to restore the initial activity. The alternatives are gasification with H_2_ or H_2_O or controlled oxidation with oxygen or oxygen-containing compounds (*e.g.*, CO_2_ itself).^341^ Gasification with H_2_O and H_2_ occurs at significant rates from 500 to 700 °C once the encapsulated material has been removed, making the metallic particles available for the catalyzed reaction.^342^ Moreover, the gasification of carbon with water was found to be faster than the one with hydrogen.^342^ Indeed, the removal of carbonaceous deposits with H_2_, like the one with CO_2_, involves dissociative adsorption and a surface reaction with carbon. In this sense, it has been successfully demonstrated that the addition of different promoters such as alkali and alkaline-earth metals enhances the gasification of carbon species and increases the stability with time. These elements enhance the adsorption and dissociation of water and CO_2_, increase the rate of gasification by H_2_ and lower the carbon solubility in the active metal. ZrO_2_, commonly used as a metal support in heterogeneous catalysts, can be used as an additive to favor CO_2_ activated adsorption, thus promoting higher levels of activity for the DRM reaction and the gasification of intermediate precursors in the carbon generation.^340^ In particular, the combination of Al_2_O_3_ and ZrO_2_ yields a reduction of catalyst deactivation by coking (Fig. 9). Although oxygen is the most effective in removing carbon deposits, a loss of activity following the regeneration process has been observed due to the oxidation of the active phase and to the loss of metallic atoms.^327^

For industrial applications, it is important to significantly improve the stability of the catalyst and make it resistant to coke. In this view, both active phase and support optimization is needed. Particle size, support defects, particle composition, and temperature-induced aggregation are the principal causes of catalyst instability. Precious metals (*e.g.*, Ru, Rh) exhibit superior activity but are limited and expensive, and thus R&D efforts have been shifted towards abundant and active metals like Ni.^92,94,96,222,343,344^ To increase the catalyst performance while retaining its stability and resistance to coke formation, several approaches such as carbon gasification^221,345–347^ through introducing redox or basic materials, alloying active metals (*e.g.*, Ni) with other metals,^228,348–351^ and enhancing the thermal stability of active metal particles by strong interaction with the support or confinement inside stable structures^352–355^ have been attempted. In order to reduce coke formation and improve catalyst stability/activity, numerous strategies involving modification in the catalyst surface composition and metal–support interaction have been attempted.^219,220,222,356–368^ To develop a coke-resistant catalyst, approaches based on selective blockage of the defect sites of the active metal (*e.g.*, Ni) nanoparticles by the use of inert elements such as S,^330^ Sn,^369^ Au,^370^ and K (ref. 234 and 371) were attempted. Atoms of these elements are supposed to be favorably localized on the defect sites of the Ni surface. For instance, Juan-Juan *et al.*^371^ have summarized the role of K in improving the stability of Ni metal with the following assumptions: (i) introduction of a small portion of K can increase the reducibility of Ni species as it enhances the interaction of NiO with the alumina support; (ii) K migrates from the support to the Ni metal surface and neutralizes a portion of active sites; (iii) coke formed during the reaction gets gasified by K without any change in its structure; (iv) K has no role in the modification of particle size or the structure of the catalyst. In an analogous study, elements such as K, S, and Au have shown improved catalytic activity by selectively blocking the active sites of the Ni stepped facet such as Ni (211),^332^ which are responsible for coke formation.^372^ Similarly, Chen *et al.*^373^ observed a promotional role of a B-based catalyst with improved coking resistance during the experiment for partial oxidation of methane (POM). Moreover, boron was suggested for effectively blocking the subsurface sites and avoiding carbon diffusion into the bulk, which compels carbon atoms to remain on the surface and react.^374,375^ Most recently, Song *et al.*^218^ developed a new technique for the preparation of Ni–Mo/MgO catalysts, wherein Mo doped Ni nano-crystallites move towards the edges of the MgO support, unite, and then stabilize at the step edges. Their catalyst exhibited high conversion and strong catalyst stability (850 hours) under DRM reaction conditions with anti-sintering and anti-coke properties.

## 4. Kinetic models

The data obtained from the reaction kinetics are applied in designing reactors, studying reaction mechanisms, and elucidating reaction–property relationships of catalyst materials. In general, the power law model is the simplest and can provide a satisfactory description of the process through a rough estimation of the required parameters.^239,376^ However, it does not account for the underlying reaction mechanisms. The Eley–Rideal (ER) model^377^ assumes that one reactant (*e.g.*, CH_4_ or CO_2_ in DRM) adsorbs onto the catalyst surface, whereas the other one reacts from the gas phase. Then, the Langmuir Hinshelwood–Hougen–Watson (LHHW) model is the result of a reaction mechanism that implies (i) the adsorption of the reactants on the active sites, (ii) their reaction at the catalyst surface, and (iii) the desorption of the reaction products, and it is based on the hypothesis that there is one (or more) reaction step slow enough to be rate-limiting (*i.e.*, the RDS), while the other ones are pseudo-equilibrated. More detailed modeling comes from microkinetic modeling (MKM), a powerful tool for the description of catalytic processes with an atomistic level of detail. A microkinetic model is a detailed kinetic model in which the single elementary steps of the reaction mechanism are characterized by their thermodynamic (*e.g.*, binding energies of reaction intermediates) and kinetic properties (*e.g.*, activation energies). The integration in time of a microkinetic model allows for the rational understanding of the reaction mechanisms occurring at the catalyst surfaces, the identification of preferred reaction paths, and corresponding active sites and RDSs. A great breakthrough was achieved with the possibility to combine DFT calculation with MKM.^230,378–380^ This powerful combination can elucidate different aspects of the reaction mechanism and the reaction kinetics without *a priori* assumptions compared with the traditional experimental kinetic methods.^372,381^

### 4.1 Reverse water-gas shift (RWGS)

The kinetics of the RWGS reaction has been widely investigated in the literature as a stand-alone process for the conversion of CO_2_ to syngas^151,382–384^ and as an inevitable side reaction in reforming processes.^144,160,385–389^ The RWGS kinetics over CuO/ZnO/Al_2_O_3_ was studied by the use of both differential and integral plug flow reactors by Ginés *et al.*^390^ A good agreement between the experimental and calculated data was obtained under different conditions by considering a Langmuir–Hinshelwood redox mechanism on Cu. However, in agreement with other authors who studied Cu-based catalysts, they noticed that the activity and the order of reaction of CO_2_ and H_2_ depend strongly on the reactants' partial pressures. This is due to a recontraction process that affected the catalyst surface caused by H_2_-rich working conditions, as proposed by Campbell and Ernst^391^ and then confirmed by many studies over Cu single-atom catalysts. Chen *et al.*^392^ performed a kinetic study of the RWGS reaction over silica-supported Cu-nanoparticles and proposed a Langmuir–Hinshelwood model starting from a formate mechanism. Kim *et al.*,^248^ on the other hand, derived two different reaction rates for the conversion of CO_2_ to CO according to associative and redox mechanisms. The initial reaction rate of the two mechanisms was consistent with the experimental data under low and high H_2_ partial pressures. However, only the redox-derived rate was able to fit the experimental data under moderate hydrogen partial pressures. Wolf *et al.*^393^ used a commercial Ni catalyst to study the RWGS reaction and determine its intrinsic kinetics to design an industrial plant. The intrinsic kinetics was examined at low residence time in a differential packed bed reactor and then modified to consider pore and external diffusion limitations. Then, the kinetic model was used in the simulation of a 1D fixed-bed reactor and validated with experimental data. Several authors used microkinetic models already developed for other processes (*i.e.* steam reforming, CO_2_ methanation, and CO_2_ hydrogenation to methanol) to gain new insights into the RWGS reaction system.^376,394^ A detailed multi-step heterogeneous reaction mechanism developed for SRM, partial and total oxidation of methane, and RWGS, for Ni-based catalysts was used by Benzinger *et al.*^395^ to interpret the experimental data obtained from the RWGS reaction in a monolith reactor. The microkinetic model, which included 42 reactions, 7 gas-phase species, and 12 surface species, was adjusted for thermodynamic consistency in the temperature range used for the study and validated with data derived from isothermal experiments in a fixed bed reactor over a commercial Ni/Al_2_O_3_ catalyst. Maestri *et al.*^161,162,237^ refined their first microkinetic model on Rh guided by the DFT-based analysis of the WGS/RWGS pathways and based on a comprehensive set of isothermal experimental data. Their semiempirical microkinetic model refined with DFT calculations was used to quantitatively describe the roles of WGS and its reverse in catalytic partial oxidation of methane on a Rh-based catalyst and it was extensively validated with experiments.

The structure-dependent microkinetic model of Cheula and Maestri^112^ describing WGS and RWGS on Rh/Al_2_O_3_ represents a novel methodology for the simulation of structure and activity of catalyst materials, allowing for the identification of the “nature” and “identity” of the active site in a self-consistent manner. In their model, the morphology of heterogeneous catalyst nanoparticles – that represents the “nature” of the active sites – is calculated using *ab initio* thermodynamics and Wulff–Kaishew construction methods. The reaction rates – that determine the “identity” of the dominant active sites – are calculated by integrating a DFT-based microkinetic model describing the catalytic activity of the crystal facets exposed by the catalyst under reaction conditions. Their microkinetic model well reproduced experimental kinetic data and reaction orders and allowed for a concomitant description of the nature of the catalyst material under reaction conditions and of its catalytic consequences in terms of reactivity.

### 4.2 CO_2_ methanation

CO_2_ methanation is thermodynamically favorable at low temperatures. However, it is limited by kinetics that strongly depends on the selected catalyst. Over the past few years, many kinetic expressions of CO_2_ methanation over different catalysts have been published.^394,396–401^ Some of them were based on simple power law models,^402,403^ while others followed more complex kinetic models.^404–407^ Most of these models are empirical and do not take into account the approach to equilibrium. Moreover, their parameters were estimated at low CO_2_ conversions and atmospheric pressure, thus far from the conditions of industrial interest. One of the first detailed mechanistic models was proposed by Weatherbee and Bartholomew^408^ for the kinetics of a 3% Ni/SiO_2_ catalyst. However, in their experiments, the gas composition was highly diluted and far away from the one required for direct injection in the gas grid without further purification or separation steps. An important breakthrough was achieved by Kai *et al.*^409^ They performed kinetic studies at atmospheric pressure over a La_2_O_3_ promoted Ni on alumina catalyst by using both a differential and an integral reactor. With an integral reactor operating at high CO_2_ conversion, they develop a Langmuir–Hinshelwood rate equation based on the mechanism proposed by Weatherbee and Bartholomew,^408^ resulting in a model able to describe in detail the influence of the reaction's products on the kinetics. Lunde and Kester^165^ proposed an empirical model over a Ru-based catalyst potentially able to predict the catalyst activity from differential to thermodynamically limited CO_2_ conversion. The original expression was derived using a fitting procedure made by the authors to data collected under differential conditions at atmospheric pressure. The same model had been used by other authors to fit experimental data collected in a larger range of CO_2_ conversions and higher pressures by modifying the kinetic parameters. Falbo *et al.*^410^ derived a novel kinetic rate equation from the one proposed by Lunde and Kester to account for the negative dependence on the partial pressure of water, improving the model capability to simulate the catalyst performance in a wide range of process conditions. The kinetics of CO_2_ methanation over a 10% Ru/γ-Al_2_O_3_ catalyst were investigated by Duyar *et al.*^411^ using a differential reactor at atmospheric pressure to obtain an empirical rate equation consistent with an Eley–Rideal mechanism where gas phase H_2_ reacts with surface species resulting from the adsorption of CO_2_. This implies that excess H_2_ would be required to boost the reaction rate. Avanesian *et al.*^412^ developed a mean-field microkinetic model for the Sabatier reaction based on DFT calculation on Ru(0001) which consisted of 18 elementary steps and was able to successfully predict the experimental data at different temperatures and reactants' partial pressures. However, this model was not able to predict the thermodynamic equilibrium conversion at different temperatures. Raghu and Kaisare^413^ proposed a mean-field microkinetic model for CO_2_ methanation at atmospheric pressure over a Ru-based catalyst by using a bottom-up modeling strategy. According to the authors, the model provided a reasonably good prediction of CO_2_ conversion within the temperature range of interest for the methanation reaction as well as under equilibrium conditions.

### 4.3 CO_2_ hydrogenation to methanol

The kinetic modeling of methanol synthesis from CO_2_/H_2_ mixtures using industrial Cu-based catalysts had been widely studied in the literature.^268,311,414–418^ Although it is generally accepted that methanol is primarily formed *via* CO_2_ hydrogenation (formate route), the role of the active sites and the effect of different catalyst components are still under debate. A variety of global kinetic models were published in the past years.^317,419–422^ Power laws and LHHW models were widely used to describe methanol synthesis, first from CO and then from CO_2_ as the main carbon source. Graaf *et al.*^423^ and Vanden Bussche and Froment^424,425^ were among the first authors to propose an LHHW kinetic model for CO_2_ hydrogenation to methanol over a commercial Cu/ZnO/Al_2_O_3_ catalyst. The main difference between the two models was the active sites involved in the activation of reactants. According to Vanden Bussche and Froment, both H_2_ and CO_2_ adsorb on the same type of active site (Cu). The first microkinetic model was proposed by Askgaard *et al.*^426^ from results obtained in surface science studies over a Cu(100) single-crystal catalyst and then successfully extrapolated for industrial conditions. Ovesen *et al.*^427^ proposed a detailed microkinetic model for methanol synthesis based on the experimental evidence collected by *in situ* EXAFS. This “dynamic” model was able to describe the change in particle morphology with the change in reaction environment as well as the reaction rate over the three basal Cu surface planes of a Cu/ZnO catalyst, both crucial features to describe the kinetic data measured under industrial conditions. Grabow and Mavrikakis^312^ conducted an extensive set of periodic, self-consistent DFT calculations over the Cu(111) facet to fit a mean-field microkinetic model to published experimental methanol synthesis rate data under realistic conditions over a commercial ternary Cu catalyst. This model included novel reaction intermediates which allowed for the formation of formic acid, formaldehyde, and methyl formate as by-products. A statistical kinetic model was derived from first-principles density functional theory (DFT) calculations and kinetic Monte Carlo simulations by Tang *et al.*^121^ over a Cu/ZrO_2_ catalyst. Despite the complexity of the reaction system, they were able to model the metal/oxide interface and demonstrate that methanol was produced through both RWGS and formate routes, assessing the contribution of each reaction channel in terms of the reaction rate. In agreement with Tang *et al.*,^121^ Ye *et al.*^428^ identified in the metal/oxide interface the most active site for CO_2_ adsorption and hydrogenation. By combining DFT calculations and microkinetic modeling to study CO_2_ to methanol conversion over a Pd_4_/In_2_O_3_ model catalyst, they demonstrated the dynamical nature of the structure of the supported Pd_4_ cluster, which transforms in response to the presence of OH in the reaction environment, modifying the main reaction pathway. Chiavassa *et al.*^429^ modeled the synthesis of methanol from a CO_2_/H_2_ mixture on a Ga_2_O_3_–Pd/SiO_2_ catalyst by combining kinetic information with relevant spectroscopic FT-IR data. They proposed a detailed reaction scheme for the CO_2_ hydrogenation bifunctional mechanism where reactants' activation occurred on two different active sites, serving as a basis for the development of an LHHW type kinetic model, able to predict the negative influence of CO due to the competitive adsorption with H_2_ on Pd sites.

### 4.4 Dry reforming of methane (DRM)

Different kinetic mechanisms for the dry reforming reaction have been published in the last few years.^224,430–435^ Only a few reports are present in the literature employing the Eley–Rideal (ER) model for DRM on Ni-based catalysts.^377^ The Langmuir Hinshelwood–Hougen–Watson model (LHHW) has more extensive applications and reports in the literature.^436,437^ Mhadeshwar *et al.* and Maestri *et al.* developed a C_1_ semiempirical microkinetic model for the conversion of CH_4_ to syngas on a Rh-based catalyst applying a hierarchical multiscale approach and first-principles calculations.^162,438^ Both were able to quantitatively predict the behaviors of several C_1_ reaction systems, including SR, DR, and CPO under different reaction conditions. Delgado *et al.*^439^ proposed a different multistep surface kinetic model on a Ni-based catalyst, consisting of 52 elementary steps with 14 surface and 6 gas-phase species. The mechanism was implemented into a 1D model of a fixed bed reactor that is able to describe the conversion of methane with oxygen, steam, and CO_2_ as well as methanation, WGS, and carbon formation *via* the Boudouard reaction. Their model has been derived by comparison of numerical simulations with data derived from isothermal experiments in a packed bed reactor, using different inlet gas compositions and operating temperatures (up to 900 °C). Aparicio^440^ derived a microkinetic model for methane reforming over Ni catalysts with many parameters obtained either from the surface science literature or from fitting the results of transient kinetic experiments. However, the model's predictions were not quantitative, as predicted rates can deviate from the experimental ones, but it could correctly predict activation energies, reaction orders, and the major trends for several reactions, such as steam and dry reforming, CO and CO_2_ methanation, WGS and RWGS. Foppa *et al.*^225^ presented an *ab initio* mechanistic study of DRM over the flat (111) surfaces of Ni, Pd, and Pt for the development of a microkinetic model made of 16 elementary steps. They provided new insights into intrinsic catalytic activity over the metallic surfaces and evaluated the interplay among all the competitive reactions that can occur under DRM experimental conditions (*i.e.*, SRM, RWGS, methane cracking, and Boudouard reaction). Based on experiments conducted in an oscillating microbalance reactor, Chen *et al.*^441^ were able to study the catalyst deactivation due to carbon formation during DRM and proposed an advanced microkinetic model for methane reforming, carbon formation, and deactivation over a Ni-based catalyst. A good agreement with the experimental kinetic data during dry reforming over fresh catalyst and with time on stream was obtained. However, the quantitative description of the deactivation process that affects reforming catalysts due to carbon formation is still an issue. The formulation of a structure-dependent microkinetic model able to simulate carbon formation under DRM conditions and its effect on the overall catalyst activity represents an intriguing challenge for heterogeneous catalysis and scientific research.

## 5. Reactor technologies

In CO_2_ conversion technologies, the design of chemical reactors is very important for its key involvement in controlling both the thermodynamic and kinetic aspects of the processes. Significant efforts have been undertaken to enhance the catalytic activity on various reactor systems starting from fixed to fluidized beds and then to multistage reactors.^57,442^

The reactions considered in this work are both endothermic (*e.g.*, DRM, RWGS) and exothermic (*e.g.*, CO_2_ methanation, CO_2_ hydrogenation to methanol). Thus, the reactor design faces different problems depending on the reaction considered but presents similarities when dealing with heat management. Exothermic reactions present heat dissipation and possible hotspot problems. The classic design of fixed bed reactors utilized in industry for several years, such as multistage adiabatic reactors and multi-tubular fixed bed reactors, presents a convenient mechanical design, low maintenance cost, high productivity per unit of volume, and limited residence time distribution of reactants and products in the reactor, in addition to the secular know-how in the construction and management. Nevertheless, such reactors present large axial and radial temperature gradients with possible hotspots, which are important issues.^443^ Recently, multi-tubular shell cooled reactors typically used in industry have been studied for the methanation reaction, with interesting results concerning the optimization of the operating conditions.^444^ The interest in structured reactors is growing due to good heat management performances, high surface-to-volume ratio, and more efficient use of the catalyst. The use of microchannel reactors provides an interesting solution for the management of heat transfer and also for improved gas/solid contact compared to traditional reactor design.^445,446^ Packed bed foams show a promising role in intensifying processes and improve the heat management of strongly exothermic/endothermic reactions, with fundamental studies that are paving the way towards the improvement in the design of such reactors.^447,448^ The use of highly conductive Ru/Ce_*x*_/Ni foams has been investigated for the methanation reaction, demonstrating promise for the management of highly exothermic catalytic reactions.^449^ Depending on the materials used for the structure and/or for the catalysts, different techniques exist and should be approached to identify the best one, with possible clogging and material coating feasibility that must be taken into account.^450^ Fluidized bed reactors are another technology already used in some industrial processes, due to the almost perfect heat management, with small or even negligible temperature gradients, thanks to the fluidization of the bed. Nevertheless, such technology suffers from the intrinsic complexity of the management of fluidization and of the broad residence time distribution. In this view, several efforts are made to increase the know-how of such technology employing advanced CFD simulations^451^ and improve their design in CO_2_ hydrogenation processes.^452,453^ Recently, plasma reactors have attracted attention in the hydrogenation of CO_2_, mainly because of their capability to run the reaction at low temperatures.^187^ This technology can involve electrically-based plasma^454^ or the addition of plasma in the gas-phase.^455,456^

Endothermic reactions present heat supply problems, which can result in the formation of cold spots in the reactors and decrease the process performance. Such problems are usually addressed by technologies similar to the ones for exothermic reactions, where the heat is usually supplied by boilers. Recently, new solutions to provide heat to the chemical reactions have been proposed, including the use of microwaves^457^ and electrified reactors.^458,459^ The electrification of reactors is a very promising technology, and it has been investigated both for the steam and dry reforming of methane,^458,459^ showing good energetic efficiency, increasing catalyst utilization, and reducing the thermal gradient in the reactor. Steady and consistent power supply, thermo-mechanical and physical properties of materials, and energy efficiency represent the main challenges when considering electrified reactors.^458–460^

**Fig. 10:**
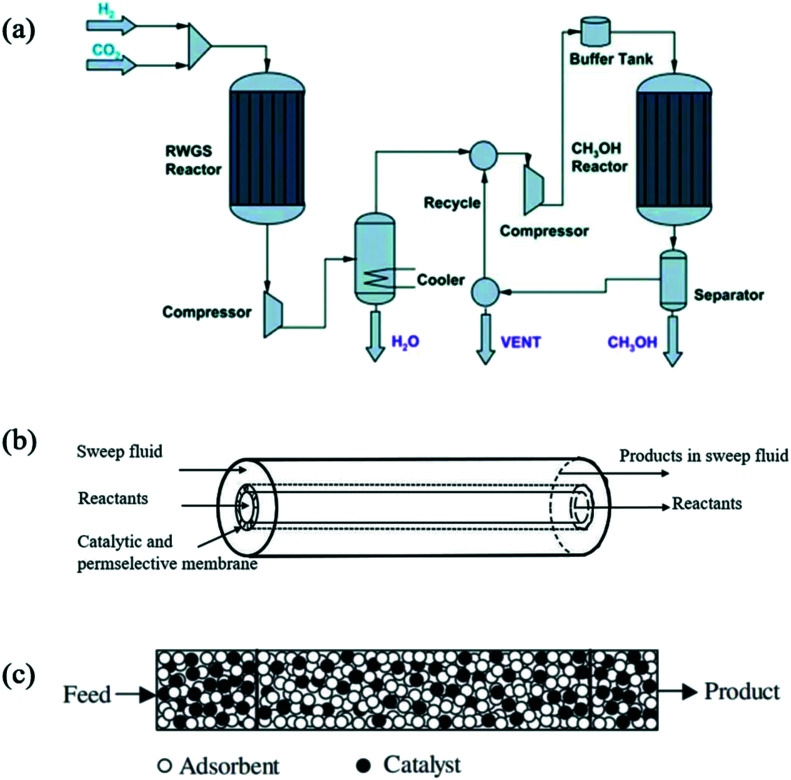
Possible solutions to suppress thermodynamic equilibrium limitations in CO_2_ conversion reactions. (a) CAMERE process (carbon dioxide hydrogenation to form methanol *via* a reverse-water-gas-shift reaction). Reproduced with permission from ref. 88. (b) Membrane reactor. (c) Sorption enhanced reactor. Reproduced with permission from ref. 461.

Besides heat management, thermodynamics is an intrinsic limit of some of these processes. To increase thermodynamic equilibrium yields, suitable pressure and temperature should be chosen, but convenient designs help to achieve good yields with less severe operating conditions (*e.g.*, lower pressure). Product removal is an increasingly feasible opportunity with several solutions proposed. The separation of CO production and subsequent methanol production has proven to be a feasible technology, with the intermediate separation of water with a separating unit (Fig. 10.a).^88,462^ Selective membranes^187,463^ are a promising technology to separate water to push the yield beyond thermodynamic limitations (Fig. 10.b). Polymeric membranes can be employed but suffer from problems at high temperatures and pressures.^464^ Zeolite membranes seem to offer a valid alternative, overcoming such problems, being stable at relatively high pressures and temperatures, and showing a relevant yield increase compared to traditional reactors.^465–467^ Stable and efficient materials for membranes at process temperatures, pressures, and compositions represent a challenge for membrane reactors.^58^ Moreover, separating water is challenging due to the similar kinetic diameters of H_2_O, CH_3_OH, CO_2_, and H_2_ (0.30 nm, 0.38 nm, 0.33 nm, and 0.29 nm, respectively).^468^ Sorption enhanced processes (Fig. 10.c) are another breakthrough technology, allowing thermodynamic limitations to be overcome by the use of an adsorbent material that removes water from the reaction environment, and have applications either in methanol production or in higher alcohols production.^469,470^ The adsorption of water simulating a complete process proved to increase the methanol productivity by 15% under practically relevant operating conditions.^471^ Recently, sorption enhanced methanol production from CO_2_ hydrogenation has provided outlet methanol concentrations up to 290% of the one without the sorption enhancement effect at 60 bar.^472^ To improve methanol production from carbon dioxide at low temperatures, magnetic field-assisted reactors are being investigated, showing positive effects on CO_2_ conversion and selectivity towards methanol.^187,473^ The presence of carbon-producing reactants and/or intermediates and/or products is another trivial challenge for some of these reactions (*e.g.*, DRM) as mentioned above. Even if catalyst design is the key to overcome such a problem, the operating conditions must be properly chosen to find the optimum between yield and catalyst deactivation, to maximize the profitability of the process.

## 6. Summary and outlook

CO_2_ is an essential molecule for our life on Earth. It plays a crucial role in the carbon-cycle which is at the basis of the mechanism of storing solar energy in chemical bonds. Moreover, thanks to its green-house properties, it affects the thermoregulation of the Earth. Without the presence of CO_2_ in the atmosphere, the average temperature of the planet would be considerably lower than the values required for life and the environment as we know them. The intensive use of fossil fuels made in the last two centuries has allowed a dramatic improvement in the quality of life thanks to the availability of enormous quantities of energy at high energy and power densities. On the flip side of the same coin, the exploitation of the energy “trapped” in the chemical bonds of the fossil fuels has released the CO_2_ originally used in the photosynthesis process to store the solar energy in the chemical bonds. As a result of these processes and together with the rapid growth of production of materials such as cement, iron, and steel (CO_2_ intensive processes), a rapid increase of the concentration of the CO_2_ in the atmosphere from 300 pm (in the pre-industrial period) to values higher than 400 ppm has been observed. The IPCC demonstrated that this higher concentration of CO_2_ can be explained with 95% confidence only by accounting for the anthropogenic activities of the last two centuries. As such, the increase of the temperature of the Earth with all the consequent effects on our life and the environment is directly linked with human activities.

Therefore, policymakers are taking action to achieve net-zero CO_2_ emissions. Solving the problem by simply eliminating the need for fossil fuels as an energy source is not straightforward since fossil resources are the basis of our current chemical and energy industry. Moreover, they are difficult to replace due to their huge energy densities. A promising concept is to replace the fossil feedstocks currently used in the chemical and energy industry with sustainably produced base chemicals and fuels by reducing CO_2_ using renewable energy (*e.g.*, H_2_ from water electrolysis with electricity from wind or solar energy – “green hydrogen”) for the production of commercially important fuels and chemicals by means of electrocatalysis, photocatalysis, or thermal catalysis routes. This would result in a carbon-neutral technology of energy transformation and storage, since CO_2_ consumption and production would occur at comparable characteristic times.

In this review, we have reported an in-depth survey of the state-of-the-art mechanistic and multiscale aspects of CO_2_ conversion to C_1_ products *via* thermo-catalysis. Though there are many chemicals in the domain of CO_2_ activation, hydrogenation products such as CO (RWGS and dry reforming routes), methane (methanation reaction) and methanol are industrially and economically highly relevant.

Catalyst design requires in-depth understanding of the reaction mechanism and surface characterization techniques. However, due to the formation of multiple products and lack in development of effective *in situ* probing techniques for thermocatalytic CO_2_ hydrogenation, elucidating the reaction mechanism and separation of product mixtures are difficult. Insights from different techniques have been reported, spanning from theoretical calculations to spectroscopy studies and kinetic investigations. We reviewed the main materials used to thermo-catalytically activate CO_2_ (a very thermodynamically stable molecule) and the main conclusions and hypothesis on the activation mechanisms and elementary pathways. The systematic understanding of the reaction mechanism with key intermediate formation and identification of surface-active sites of the catalysts are of great help in establishing the CO_2_ activation process and obtained desired conversion. A significant role of the catalyst's shape was also reported. This finding could lead to rational design of catalysts and judicious plans of new nano-catalysts for potential use in carbon dioxide hydrogenation. Particular attention has been paid to catalyst deactivation due to coke formation that is a major technological and scientific challenge, requiring innovations in materials and process design to combat. Deactivation is mainly caused by filamentous carbon as it has high mechanical strength that leads to mechanical deformation of the catalyst and blocks metal active sites. We then reviewed the main kinetic schemes reported in the literature (both detailed microkinetic models based on first-principles calculations and rate equations): the main open area of progress for kinetic modeling in this context is to couple the description of the kinetic events with coke formation and its kinetic consequences. Design, innovations, and engineering of catalytic materials including reactors and process technologies are vital solutions, but such approaches are yet to be established for commercial success. Integrating reactor design and developing more effective and selective catalysts may solve scientific and technological challenges by reducing the activation barrier in an energy efficient process.

## Conflicts of interest

There are no conflicts to declare.

## Supplementary Material
